# The Keio Medical Science Prize for 2015

**DOI:** 10.1038/npjamd.2016.13

**Published:** 2016-04-07

**Authors:** Kazuo Tsubota

**Affiliations:** 1 Department of Ophthalmology, Keio University School of Medicine, Shinjuku-ku, Tokyo, Japan

Understanding mechanisms that underlie disease holds a key to healthy aging if the diseases in question are age-related. This field that is already very broad is continually expanding as new phenomena or biological processes are shown to affect healthy aging. The 2015 Keio Medical Science Prize (http://www.ms-fund.keio.ac.jp/prize/), which since 1996 has been awarded annually for outstanding contributions to clinical medicine, acknowledged the gut microbiome and autophagy, both of which have a pivotal role in health and disease. The two laureates were Jeffrey I. Gordon from Washington University School of Medicine in St Louis and Yoshinori Ohsumi of Tokyo Institute of Technology, respectively. Thanks to the pioneering work of Gordon, we now know that tens of trillions of microbes live in the human gut, where they process food and synthesize vitamins and nutrients that our human cells cannot make. Gordon’s work has changed the understanding of the most important health problem—obesity—which is the key target condition to ensure healthy aging. His work, as well as that of many others, supports the hypothesis that dysfunctional gut microbiome is an underlying cause of obesity, making us extend our focus beyond food intake and genetic predisposition. It is increasingly clear that the food-microbiome interaction is the key to successful health from the perspective of nutrition. As the editor-in-chief of *npj Aging and Mechanisms of Disease*, I would like to specifically invite original research submissions which shed further light on the role that the human microbiome has in aging and disease.

Ohsumi’s work also opened the new field of science and autophagy. Using a yeast genetic approach, he discovered the molecular mechanism of autophagy and the biological process, whereby cells degrade their cellular components and subsequently reuse them. Over the course of this career, he identified 15 autophagy-related genes and characterized their functions. Autophagy is not only related to various conditions, such as physiological development and antigen presentations, but it is also important in neurodegenerative diseases and the most prevalent of age-related diseases of them all—cancer. I feel very proud that a Japanese scientist contributed to such a significant field of biological science and expanded its breadth in the research field. Autophagy is another area I would welcome original research submissions that expand on the work done by Ohsumi.

Many of the previous awardees of The Keio Medical Science Prize have made important contribution to aging science. In 2013, one of the laureates was Shigekazu Nagata who received this award for his work on apoptosis and its role in physiology. In 2012, the award was given for immunotherapy in cancer to Steven Rosenberg and to Hiroyuki Mano for elucidating the role of anaplastic lymphoma kinase (ALK) deregulation in lung cancer and the power of ALK inhibitors in treating this disease. In 2009, Jeffrey Friedman received the prize for his work on leptin and elucidating its role in appetite and adiposity regulation. Going further back, Fred Gage was awarded the prize for his discovery of neurogenesis in the adult. All of these discoveries and the follow-up work fall firmly within the scope of *npj Aging and Mechanisms of Disease*.

The 2016 Keio Medical Science Prize nominees are now under consideration. It is my hope that the prize is again awarded within the expanding field of age-related medicine.

